# Recent Progress in Antioxidant Active Substances from Marine Biota

**DOI:** 10.3390/antiox11030439

**Published:** 2022-02-22

**Authors:** Todorka Vladkova, Nelly Georgieva, Anna Staneva, Dilyana Gospodinova

**Affiliations:** 1 Laboratory for Advanced Materials Research, University of Chemical Technology and Metallurgy (UCTM), 8 “St. Kl. Ohridski” Blvd, 1756 Sofia, Bulgaria; ani_sta@mail.bg; 2 Department of Biotechnology, University of Chemical Technology and Metallurgy (UCTM), 1756 Sofia, Bulgaria; neli@uctm.edu; 3 Department of Electrical Apparatus, Technical University of Sofia, 1756 Sofia, Bulgaria; dilianang@tu-sofia.bg

**Keywords:** natural antioxidants, marine sources, antioxidant substances, derivation technologies

## Abstract

Background: The well-recognized but not fully explored antioxidant activity of marine-biota-derived, biologically active substances has led to interest in their study as substitutes of antibiotics, antiaging agents, anticancer and antiviral drugs, and others. The aim of this review is to present the current state of the art of marine-biota-derived antioxidants to give some ideas for potential industrial applications. Methods: This review is an update for the last 5 years on the marine sources of natural antioxidants, different classes antioxidant compounds, and current derivation biotechnologies. Results: New marine sources of antioxidants, including byproducts and wastes, are presented, along with new antioxidant substances and derivation approaches. Conclusions: The interest in high-value antioxidants from marine biota continues. Natural substances combining antioxidant and antimicrobial action are of particular interest because of the increasing microbial resistance to antibiotic treatments. New antioxidant substances are discovered, along with those extracted from marine biota collected in other locations. Byproducts and wastes provide a valuable source of antioxidant substances. The application of optimized non-conventional derivation approaches is expected to allow the intensification of the production and improvement in the quality of the derived substances. The ability to obtain safe, high-value products is of key importance for potential industrialization.

## 1. Introduction

Marine organisms and their metabolites are the focus of worldwide efforts for the discovery of novel, biologically active products that are accepted as a fundament of the future bioeconomy and especially of promising drugs. In some cases, the biological activity of marine-organism-produced, unusual, structurally diverse compounds is higher than that of substances from terrestrial sources [1]. Exposed to environmental stresses and changes, the marine biota develops protective mechanisms, leading to the formation of secondary metabolites and macromolecules with antioxidant activity that is largely recognized [2]. The identification of marine antioxidants and their potential applicability in industries such as pharmaceutical, nutraceutical, cosmetic and others have attracted extensive research interest [3,4,5,6,7,8,9,10,11,12,13,14]. Many antioxidant substances—peptides, polyphenols, polysaccharides, carotenoids, etc.—display additional biological functions: antimicrobial, anticancer, anti-diabetic, anti-Alzheimer, anti-fibrotic, neuroprotective, sleep-enhancing, lipid-lowering, wound healing and skin protection [15]. The increase in chronic infections, often associated with colonization by drug-resistant pathogens, increases the need for dual-active compounds with antioxidant and antibacterial activities combined in one molecule [16].

A large variety of marine organisms and their metabolites are reported to deliver antioxidant products in the form of biomass, crude total or sequential extracts and pure substances [17]. Despite the number of reports about both in vitro and in vivo antioxidant studies of extracts, fractions, synergistic mixtures and single compounds, the search for marine sources, broad-spectrum substances with antioxidant activity and new properties, interesting for a variety of industries, remains of great interest. It is expected that non-conventional, optimized derivation will provide sustainable alternative techniques to preserve the potency of extracted antioxidant compounds, together with many other benefits [18].

This review is an update for the last 5 years on the marine sources of natural antioxidant substances, different classes of antioxidant compounds, current derivation biotechnologies and characterization techniques, aimed at the presentation of the current natural marine antioxidant products for potential industrial applications.

## 2. Oxidation and Antioxidants

**Figure 1:**
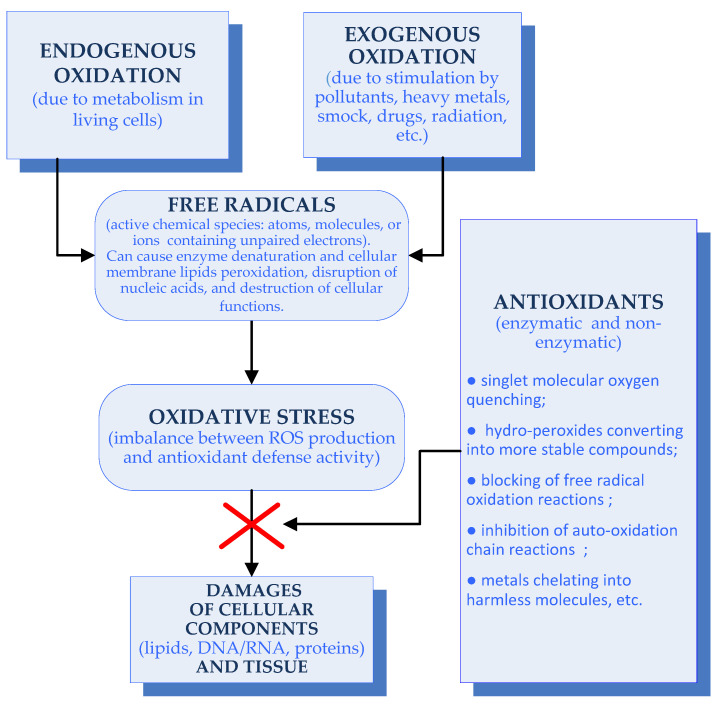
Simple sketch of oxidation process and effects of antioxidants.

The oxidation process is a chemical reaction, involving the transfer of hydrogen or oxygen atoms or electrons, free radical production and chain reactions, causing tissue injuries [19]. A simple illustration of this process and the effects of different antioxidants is presented in Figure 1.

Free radicals are reactive chemical species (atoms, molecules, or ions) that usually contain unpaired electrons. They are produced endogenously due to metabolism in living cells, or exogenously due to stimulation by pollutants, heavy metals, smoke, drugs, radiation, etc. Examples of free radicals are peroxides (O_2_^•−^), hydroxyl radicals (^•^OH), hydrogen peroxide (H_2_O_2_), nitrogen dioxide (^•^NO_2_), singlet oxygen (^1^O_2_), etc. Free radicals can cause enzyme denaturation and cellular membrane lipid peroxidation, disruption of nucleic acids, and the destruction of cellular functions. Such damages are termed oxidative stress [19].

Oxidative stress is an imbalance between the production of reactive oxygen species (ROS) and antioxidant defense activity. Excessive ROS can cause damage to various cellular components that leads to tissue damage, associated with aging and various chronic diseases, such as cancer, diabetes, neurodegenerative and cardiovascular diseases, and others. Natural antioxidants play remarkable roles in the inhibition of ROS production [17,20].

An antioxidant is any compound that stops the oxidation process by inhibiting a free radical reaction. In the biological systems, antioxidants prevent the damaging effects produced during metabolism, such as highly reactive free radicals, neutralizing their excess in the body. The antioxidant defense mechanisms can be divided into two main types: enzymatic and non-enzymatic. The enzymatic antioxidants include superoxide dismutase, catalase, ascorbate peroxidase, and glutathione reductase. The non-enzymatic antioxidants include organic compounds, such as peptides, polysaccharides, polyphenols, vitamins, and others [18,21,22]. Non-toxic and biodegradable marine antioxidants could be used to reduce oxidative stress by free radical scavengingapplied in a food preservation and cosmetics; cancer, autoimmune disorders, ageing and some degenerative diseases preventing, etc. [16,18,20].

The tests that are most often used for antioxidant activity evaluation include 1,1-diphenyl-2-picryl hydrazil (DPPH) radical scavenging; deoxyribose assay; ferric-reducing antioxidant power (FRAP) assay; nitric oxide (NO) scavenging, 2,2′-azino-bis-3-ethylbenzothiazoline-6-sulfonic acid (ABTS) radical scavenging; lipid peroxide inhibition of superoxide radicals, hydroxyl radical scavenging assays; and total antioxidant capacity (TAC) [17].

## 3. Marine Sources of Natural Antioxidants

The marine environment is a rich but underexploited source of commercially interesting natural products with antioxidant activity. A large variety of marine macro- and microorganisms, such as seaweeds, microalgae, sponges, ascidians, bryozoan, lichen, bacteria, fungi, etc., are sources of natural antioxidants. The antioxidant capacity depends on the marine species and the extraction and purification technologies, as well as on the operation conditions. It depends also on the climatic conditions under which the marine biota grow, which makes antioxidants from the same species compositionally different across the globe [23,24,25,26].

The identification of marine sources of natural antioxidants includes the preparation of crude total or sequential extracts from marine biota and the evaluation of their antioxidant activity as a first step. A second step is the isolation, purification, and identification of natural compounds with antioxidant activity. In some cases, a third step follows, which is the copying of natural antioxidant compounds by chemical synthesis. Many marine organisms that deliver antioxidants are already known and many continue to be identified. A trend to transform marine biota waste and byproducts into resources has been observed lately [27].

**Figure 2:**
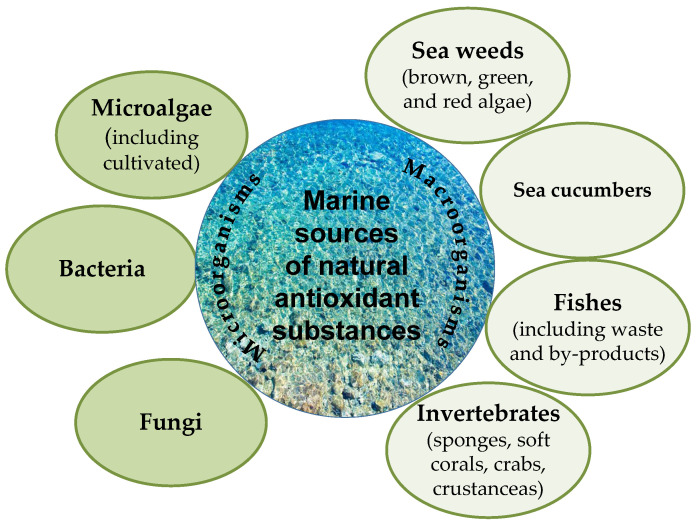
Main marine sources of substances with antioxidant activity.

This section presents marine sources (including byproducts and wastes) of natural antioxidants identified during the last 5 years by preparation and evaluation of the antioxidant activity of crude extracts from marine macro- and microorganisms, new bacterial strains, antioxidant metabolites, etc. New antioxidant activities and their potential pharmaceutical and other applications are also included. The main marine sources of natural compounds with antioxidant activity included in this review are summarized in Figure 2 and Supplementary Table S1.

It is evident that substances with antioxidant activity can be derived from a vast range of marine macro- (seaweeds, cucumbers, fishes, and invertebrates—sponges, soft corals, crabs, crustaceans) and microorganisms (microalgae, bacteria, and fungi).

### 3.1. Marine Macroorganisms

#### 3.1.1. Seaweeds (Marine Macroalgae)

Marine algae are photosynthetic organisms with simple reproductive organs that can be unicellular or multicellular and, respectively, they are named micro- or macroalgae (seaweeds). The Food and Agriculture Organization (FAO) classifies the marine macroalgae as brown—*Phaeophyceae*, green—*Phylum Chlorophyta*, and red—*Phylum Rhodophyta*, according to their pigmentation [20,28]. The marine seaweeds are considered the richest source of extraordinarily potent marine antioxidants and therefore the most studied as their sources. The cosmetic value and nutritional and therapeutic activities of marine macroalgae are presented in numerous review and research papers [17,18,29,30]. Multi-disciplinary scientific approaches including physiological, molecular, chemical, technical, and technological are expected to achieve improved marine algal factory processes [31]. The review of Tziveleka et al. [32] summarizes the antioxidant potential and biogenetic origin of 301 macroalgal metabolites, categorized according to their chemical classes, highlighting the mechanisms of antioxidative action when known. The classification, distribution, and main results of experimental antioxidant activity studies of marine algae in Egypt, as well as the use of their biomass, are presented by Rashad and Chaghaby [33].

The highest antioxidant activity of brown, followed by red and green, algae extracts is demonstrated in a number of studies [34,35]. V. Panajotova [36] confirms this by a complex study of five, scarcely investigated, most widely distributed Black sea green, brown and red algae—*Ulva rigida*, *Chaetomorpha linum*, *Gelidium crinale*, *Cystoseira barbata*, and *Cystoseira crinite*—connected to their utilization as a raw material for the food, pharmaceutical, and cosmetic industries. Miranda-Delgado et al. [37] report the highest antioxidant activity of ethyl acetate and dichloromethane extracts of *Libertia chilensis* between four brown and red seaweeds, collected from the Chilean coast. The highest antioxidant activities found in methanol and aqueous extracts of fifteen marine brown, followed by red and green, macroalgae from the Brazilian coast are another confirmation [34]. The synergetic coexistence of polyphenols and alkaloids is supposed to be a reason for the high antioxidant activity [38,39].

Brown seaweeds such as *Sargassum ilicifolium* and *Sargassum angustifolium*—Qeshm Island, Iran—[40] and *Sargassum filipendula*—Indonesia [41]—are some of the main marine sources of antioxidant compounds with antioxidant and antimicrobial activities. The pioneering research of Arguelles and Sapin [42] in the Philippines shows the potential of extracts from *Turbinaria decurrens Bory* as a cheap source of antioxidant active compounds with potential use in cosmetics and in the treatment of drug-resistant bacterial infection and diabetes. El-Sheekh et al. [43] experimentally show the high antioxidant activity—by FRAP—of the ethanol extract of *Toania atomaria* (*Phaeophyta*) from the Rocky Bay of Abu Qir in Alexandria, Egypt.

New bioactive components with antioxidant and antibacterial activities were identified in crude extracts of the seaweed *Caulerpa racemosa var. cylindracea* from the Algerian coast. Such bioactive components were not reported previously for the same seaweed from other geographical areas [44]. Methanol extracts of two thus unexplored Bangladeshi brown seaweeds, *Padina tetrastromatica* and *Gracilaria tenuistipitata*, demonstrated antioxidant activity by DPPH, ABTS, FRAP, phosphor-molybdenum, H_2_O_2_ and NO scavenging assays); values were higher for those of *Padina tetrastromatica* than of *Gracilaria tenuistipitata*, due to the higher total phenolic and flavonoid content [45]. Acyl-phloro-glucinol derivatives from brown alga *Zonaria tournefortii* demonstrated potential antioxidant activities in DPPH and ABTS assays, as found by Hamiche et al. [46]. In a comparative study, Lee et al. [47] found that both celluclast-assisted hydrolysate from conventionally used *Ecklonia maxima* blades and viscozyme-assisted hydrolysate from lesser used *E. maxima* stipe possess potent antioxidant and anti-inflammatory properties and may be utilized as functional ingredients in the food and functional food sectors. A bioactivity profile study [29]—by ABTS assay and sun protection factor—of maceration extracts from three edible brown seaweeds, *Eucheuma cottoni*, *Sargassum polycystum*, and *Caulerpa racemose*, showed that *C. racemosa* extract provides very strong antioxidant activity and it is able to protect the skin from UV exposure.

Red seaweeds are another rich source of compounds with antioxidant and various other activities: antimicrobial, antifouling, anti-proliferative, and anticancer. Ramadani et al. [48] demonstrate that the ethanol extract of red alga *Gracilaria bursapastoris*, harvested in Nador lagoon (Maroco), exhibits high antioxidant activity due to the presence of a large amount of phenolic compounds. Hmani et al. [49] report that methanol extracts of six species, *Asparagopsis armata*, *Gracilaria gracilis*, *Hypnea musciformis*, *Laurencia obtusa*, *Pterocladiella capillacea*, and *Sphaerococcus cornopifolius*, from twelve studied red macroalgae collected from the northern coast of Tunisia show significant DPPH radical scavenging activities and TAC.

#### 3.1.2. Sea Cucumbers

Sea cucumbers from different locations in the world are presented in a number of reports as an economic source of antioxidants. Highly concentrated acetonitrile/tri-fluoroacetic acid, methanol, and water/methanol extracts of one of the most harvested sea cucumbers in Turkey, *Holothuria tubulosa Gmelin 1791*, demonstrated antioxidant activity that was close to that of the reference antioxidant agents [50]. High antioxidant activities, evaluated by ABTS and DPPH assays, TAC, and FRAP, were found in the tegument extract of *Holothuria tubulosa* from the Bizerta lagoon in Northern Tunisia [51]. The antioxidant and anti-melanogenic activities of an ultrasonic extract from red sea cucumber *Stichopus japonicas* (*S. japonicas*) were reported by Ding et al. [52]. Ardiansyah et al. [53] found *Holothuria leucospilota* to be the best source of antioxidant compounds as its methanol extracts were found to possess the highest antioxidant activity (IC_50_ value of 9.66 ± 0.15 mg mL^−1^) among 16 Indonesian sea cucumbers from the genera *Actinopyga*, *Bohadscia*, *Holothuria*, *Pseudocolochirus*, and *Stichopus*. Nugroho et al. [54] found the strongest antioxidant activity (IC_50_ = 14.22 ± 0.87 µg µL^−1^) for *Holothuria atra* among 21 Indonesian sea cucumbers evaluated. Prepared by food-grade enzymes (alcalase, α-chymotrypsin, flavoenzyme, kojizyme, neutrase, papain, pepsin, protamex, and trypsin), hydrolysates of the sea cucumber *S. japonicas* possess strong antioxidant activity in vitro and in vivo against hydrogen peroxide-induced oxidative stress [55].

#### 3.1.3. Fishes

Marine fishes are also reported as a source of substances with antioxidant activity. Zhou et al. [56] demonstrate that *Raja porosa* skate cartilage (RPCS) delivers a relatively uniform polysaccharide, chondroitin sulfate (CS). This CS displays more effective free radical scavenging than shark CS. This indicates the potential of RPCS to promote oxidative stress resistance and to act as an antioxidant agent. Guedes et al. [57] used *Sardina pilchardus* roe as a lipid source to produce liposomes as carriers with antioxidants and anti-inflammatory bioactivities. Radical scavenging assays demonstrate that fish roesomes efficiently neutralize peroxyd-, hydroxyl-, and nitric oxide radicals. Kurhaluk and Tkachenko [58] found sex-related relationships between the pro- and antioxidant balance and the tissue type in the adult stage of sea trout *Salmo trutta* m. *trutta* L. sampled in the Pomerania region, Northern Poland. Modifications in the lysosomal functioning, induced by long-term environmental stress, associated with the habitats changing from freshwater to seawater and intense migrations, were observed.

#### 3.1.4. Marine Invertebrates

Marine sponges, corals, and a single bivalve, exposed to high levels of ROS in the ocean, are reported to produce antioxidant compounds as a major defense mechanism against free-radical-mediated toxicity. Ganesan et al. [59] analyzed the global market of marine invertebrates to gain more insight into their functional properties, focusing on the antioxidant, anticancer, and antimicrobial activities of derived peptides and proteins. Despite some variations, the antioxidant-producing potential of 100 different biomes, isolated in methanol extracts of sponges, corals, and the single bivalve, was high, due to the presence of antimicrobial peptides [60]. Biologically active complex substances with a potential application in liposomal drug delivery were, for the first time, derived from black sea invertebrates by two-phase extraction in combination with ultrasonication [61].

Marine sponges are some of the main sources of antioxidant substances. Muthiyan et al. [62] demonstrated that the secondary metabolites present in methanol extracts from the marine sponge *Hyrtios erectus*, collected from the North Bay of the South Andaman Sea, showed potential antioxidant and anti-inflammatory activities, but further studies are required to identify the bioactive compounds. Marine sponges of the genus *Suberea* (family: *Aplysinellidae*) are recognized as producers of bromotyrosine derivatives, exhibiting structural diversity, ranging from simple monomeric molecules to more complex molecular scaffolds with various biological and pharmacological potential [18,63]. Fahmy and Abdel-Tawab [64] experimentally showed that marine sponge *Diacarnus ardoukobae* associated *Streptomyces* sp. *NMF6* strain could produce secondary metabolites possessing several activities: antioxidant—proven by DPPH, FRAP, and phosphor-molybdenum assays—antimicrobial, anticancer, and antiviral.

**Figure 3:**
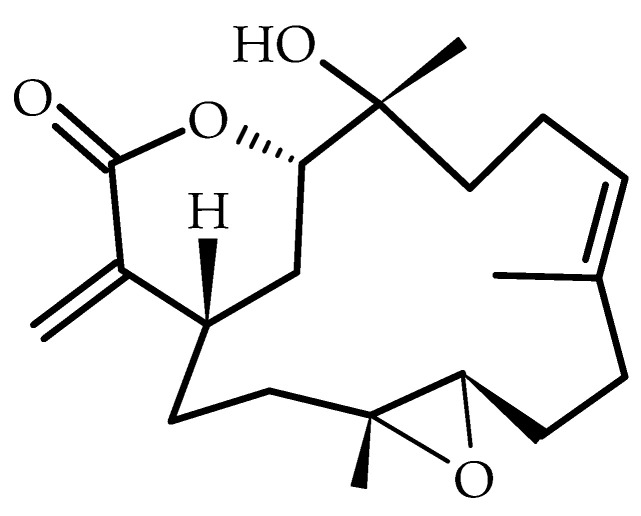
Chemical structure of sinularin.

Marine soft corals contain a variety of secondary metabolites with diverse biological activities, including antioxidant, cytotoxic, and others. Yegdaneh et al. [65] found that the evaporated methanol-ethyl acetate (1:1) maceration extracts of *White Menella* sp.—with IC_50_ values of 0.056 µg/mL—showed the highest antioxidant activity among several soft corals, namely *Junceella juncea*, *Cavernularia* sp., *white Menella* sp., *brown Menella* sp., *Virgularia* sp., *Sinularia compressa*, *Sinularia variablis*, and *Sinularia polydactyla*, collected from the Persian Gulf. Wang et al. [66] compared the antioxidant potential and the anti-proliferative effect for cancer cells of two unknown substances, sinularin (its chemical structure is given in Figure 3) and dihydrosinularin, derived from soft corals.

Both sinularin and dihydrosinularin promptly react with DPPH, ABTS, and hydroxyl radicals (•OH), demonstrating general radical scavenging activity. Both sinularin and dihydrosinularin also show the induction of Fe^+3^ reduction and Fe^+2^-chelating capacity, which strengthen their antioxidant activities. Sinularin shows higher antioxidant capacity than dihydrosinularin. The adenosine triphosphate (ATP) assay indicated a stronger anti-proliferation effect of sinularin than that of dihydrosinularin on breast, lung, and liver cancer cells [66]. Crabs are also reported to be sources of antioxidant substances. The alcalase hydrolysates of rocky shore crab *Grapsus albolineathus* contain bioactive peptides with potent antioxidant and antibacterial activities that are affected by the hydrolysis level, as was found by Shaibani et al. [67]. Yogeshwaran et al. [68] studied the bioaccumulation of antioxidants, heavy metals, and metabolic enzymes in the crab *Scylla serrata* from three different regions of Tuticorin, on the Southeast Coast of India.

Crustaceans are discussed as an excellent source of antioxidant peptides, chitin derivatives, and carotenoids for retarding lipid oxidation, causing the deterioration of marine foods [69]. The peptide profile and free radical scavenging activity of low-molecular-weight peptide fractions from whole-body extracts of two common marine mollusks, *Tympanotonus fuscatus var radula* (*Linnaeus*) and *Pachymelania aurita* (*Muller*), obtained from the Niger Delta region, indicate that they could find application as natural antioxidants [70]. The experimental results of Maduraiveeran et al. [71] suggest that nematocysts’ crude venom from jellyfish *Acromitus flagellatus* possesses well-expressed antioxidant activity: free radical scavenging potential—56.36%; DPPH—72.47% and hydroxyl radicals—68.50%; superoxide anion—65.75%. It also showed anticancer activity against A549 and HepG2 cancer cell lines. Biochemical analysis proves the presence of proteins, lipids, and carotenoids.

### 3.2. Marine Microorganisms

Many microorganisms, including microalgae, both Gram-negative and Gram-positive bacteria, fungi, actinomycetes, archaea, protozoa, and yeast, are identified as producers of natural antioxidants. The advances in research have led to the discovery of unknown microorganisms producing uncommon secondary metabolites. Marine microbial resources from the Gulf of Mannar Bay of Bengal, India, were screened for antioxidant molecule production and Tripathi et al. [72] revealed that *Kocuria marina CDMP 10* extract can effectively reduce DPPH free radicals.

#### 3.2.1. Microalgae

Microalgae are diverse organisms capable of accumulating bioactive metabolites, making them promising feedstocks for a variety of applications, such as functional foods, bio-fertilization, and others. In many cases, such metabolites combine two or more types of biological activity: antioxidant and skin-regenerative, or antioxidant, antibacterial, and anticancer, etc. Microalgal antioxidant production and the scavenging ability of such antioxidants is discussed by Sanson and Brunet [73], with a focus on microalgae-produced sterols, vitamins, and phenolic compounds. Widowati et al. [74] found that the antioxidant potential was dependent on the total phenol content—evaluated by DPPH assay—for methanol extracts from microalgae, *Dunaliella salina*, *Tetraselmis chuii*, and *Isochrysis galbana*, clone Tahiti. Wali et al. [75] demonstrated the well-expressed in vitro antioxidant, antimicrobial, and anticancer activity of microalgae *Nannochloropsis oculata* extracts, delivering terpenoids along with carotenoids, polyphenols, and fatty acids. The antioxidant and anti-inflammatory effects of the microalgae *Nannochloropsis gaditana* on streptozotocin-induced diabetes mellitus in Wistar rats were demonstrated by Nacher et al. [76]. Gϋrlec et al. [77] found that crude extracts of several microalgae, i.e., *Galdieria sulphuraria*, *Ettlia carotinosa*, *Neochloris texensis*, *Chlorella minutissima*, *Stichococcus bacillaris*, *Schizochytrium limacinum*, *Crypthecodinium cohnii*, and *Chlorella vulgaris*, showed high radical scavenging and good cytotoxic activity, both indicating the microalgae’s potential for use in novel therapeutic approaches.

Cultivated microalgae have represented a significant source of natural antioxidants during the last several years. López-Hernández and García-Alamilla [78] reported continuous microalgae cultivation in photo-bioreactors, underlining three important factors in the production of bioactive compounds: microalgae species, medium composition, and operation parameters. The antioxidant content and productivity of the microalgae were assessed in this respect. All microalgae produce tocopherols and carotenoids, except *S. platensis* and *Porphyridiumcruentum*, which produce phycocyanin and allophycocyanin. *Spirulinaplatensis*, *Isochrysisgalbana*, and *Tetraselmissuecica* produce phenols, terpenoids, and alkaloids. Archer et al. [79] phototropically grew ninety-one (91) microalgae strains (isolated from aquatic habitats in Irish waters) in nutrient-enriched media to generate biomass, which was harvested and assess for antioxidant potential. Two heterokont marine strains’ extracts, *Bacillariophyte* cf. *Stauroneis* sp. *LACW24* and *Ocrophyte* cf. *Phaeothamnion* sp. *LACW34*, demonstrated potential for biomass cultivation and valorization, important for further acceptance as a novel species within the relatively narrow range of commercially exploited marine microalgae species. Vilakazi et al. [80] showed that the cultivation of algal strain *Chlorella* sp. *S14* produced biomass for polyunsaturated fatty acid (PUFA)-rich extracts (52.87%) with antioxidant and anti-proliferative properties.

#### 3.2.2. Marine Bacteria

Secondary bacterial metabolites possess a wide range of biologically active compounds. Screening methodologies for the detection of bioactive marine bacteria, the identification of antioxidant metabolites, and their pharmaceutical applications are reported by Santos et al. [81]. The promising antioxidant and antibacterial activities of a new Gram-positive marine *Actinobacteria* strain, isolated from the unexplored sea sediment of Alang, Gulf of Khambhat, Gujarat, were presented by Dholakiya et al. [82]. Choi et al. [83] found that, among bacteria isolated in extracts of marine sediments, those of *Streptomyces* sp. *SCS525* showed strong antioxidant activity. Bioassay-guided fractionation and spectroscopic data analyses led to the identification of two antioxidant compounds, gramicidin A and gramicidin B, which are known to inhibit the polyketide type III pathway-related protein GCS and spore germination. The experimental results suggest that safe and low-cost antioxidants can be produced from marine bacteria on a large scale [83]. Baker et al. [84] found that some algae-associated bacteria from the Red Sea of Jeddah, Pakistan, produce secondary metabolites with strong antioxidant activity. Phytochemical analysis revealed the presence of steroids, saponins, tannins, flavonoids, anthocyanin, and betacyanin in all tested extracts, which indicates their possible potential use in the pharmaceutical industry. Hassan et al. [85] reported, for the first time, salinity stress exploitation to promote the production of antioxidants from bacterial isolates, which can be utilized in foods and postbiotics. It was demonstrated that the salinity stress enhanced the antioxidant capacity of *Bacillus* and *Planococcus* species isolated from the saline Aushazia Lake, Qassim region, Saudi Arabia.

Marine cyanobacteria of the genus *Chroococcidiopsis* sp., recently unveiled to go to Mars, were evaluated by Asunción et al. [86] as a valuable source for the antioxidant industry. Combined safe solvent extracts were prepared after preliminary treatment of *Chroococcidiopsis* sp. *LEGE 06174* with sulfuric acid, NaOH, PBS, DMSO 20%, DMSO 100%, and acetone. The most effective toward overall pigment extraction and dissolution of the studied mucilaginous sheath was the pre-treatment with PBS. Extracts of PBS-pre-treated *Chroococcidiopsis* sp. demonstrated a high antioxidant capacity due to the high content of polysaccharides, scytonemin, phycobiliproteins, and phenolic compounds. The highest total carotenoid content was found in methanol–PBS extracts, whereas in the ethanol–PBS extracts, the highest level of phenolic compounds was found [86].

Bacteria and microalgae are known as the most important producers of valuable antioxidant enzymes—superoxide dismutase and catalase—and antioxidant substances—carotenoids, exopolysaccharides, and bioactive peptides—with various biological properties and applications. Hamidi et al. [87] compared marine bacteria and microalgae, seeking to determine which is the best for the biotechnological production of antioxidant substances. The current knowledge about the product yield, health-related benefits, and potential applications in various biological and industrial fields is summarized in this review.

#### 3.2.3. Fungi

Fungi play a significant role in the production of secondary metabolites with interesting antioxidant properties. Evaluating forty-five (45) secondary metabolites derived from marine fungi and bacteria, Hamed et al. [88] found that all extracts of fungal isolates showed higher antioxidant activities than those of bacterial isolates. Vitale et al. [89] reviewed the methodology of fungal antioxidant production: extraction strategies, available tools for antioxidant activity evaluation, and description of various classes of marine fungi antioxidants, including the most recently discovered antioxidant compounds from endophytic fungi and mushrooms.

**Figure 4:**
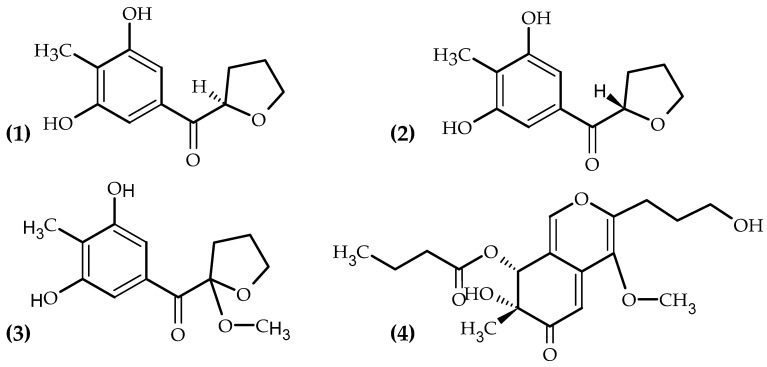
New compounds isolated from fungus *Myrothecium* sp. *BZO-L062*: (−)-(1S)-myrotheciol (**1**), (+)-(1R)-myrotheciol (**2**), 1-methoxy-myrotheciol (**3**), and myrothin (**4**).

The in vitro protective effect of the marine fungus *Aspergillus puulaauensis* TM124-S4’s extract on H_2_O_2_-stressed primary human fibroblasts was shown by Letsiou et al. [90]. The change in gene transcripts reveals that *A. puulaauensis* TM124-S4 extract exhibits skin protection properties by mediating cell proliferation, the antioxidant response, skin hydration, and DNA repair. The extracts modulate also the expression of genes involved in skin pigmentation and aging [90]. Lekshmi et al. [91] show that the less explored sponge-associated endophytic fungi are excellent sources of bioactive molecules. Evaluated by corresponding bioassays, nineteen (19) endophytic fungi, isolated (using Sabouraud Dextrose Agar Medium) in three marine sponges, *Tedania anhelans*, *Myxilla arenaria*, and *Callyspongia fibrosa*, collected along the east and west coasts of India, demonstrate antioxidant, anticancer, and/or anti-inflammatory activities [91]. Saravanakumar et al. [92] provide information on the secondary metabolites produced by marine fungi of different origin. Mangroves are presented as a fungal paradise. Several marine fungi isolated from brown, red, and green algae, as well as from soft corals and red, blue, and black sponges, are presented as having potential medicinal value. Fungal metabolites, derived from *Penicillium flavigenum*, isolated in hypersaline water, are shown to have antioxidant and anti-proliferative activities [92]. Lu et al. [93] found that deep-sea-derived fungus *Myrothecium* sp. *Bzo-l062* could be a source of four new antioxidant and anti-inflammatory components: a pair of 2-benzoyl tetrahydrofuran enantiomers, namely (−)-1S-myrothecol (1) and (+)-1R-myrothecol (2); a methoxy-myrothecol racemate (3), and an azaphilone derivative, myrothin (4). Their chemical structures are presented in Figure 4.

The new compounds (**1**) and (**2**) exhibited antioxidant activity in the ABTS assay and oxygen radical absorbance as well as anti-inflammatory activity and nitric oxide formation in lipopolysaccharide-treated RAW264.7 cells.

## 4. Antioxidant Substances from Marine Organisms

Two main types of natural antioxidant substances are derived from marine organisms: enzymatic—superoxidaze dismutase (SOD), catalase (CAT), glutatione peroxidase (GTx), and glutatione reductase (GRx)—and non-enzymatic—different classes of organic compounds and minerals. Antioxidant enzymes are considered as the first line of defense against ROS and SOD, as the most powerful antioxidants in the cells. Their daily intake protects the immune system and slows down the aging process. Some antioxidant peptides, amino acids, polyphenols, therpenoids, etc., are already in use in the cosmetics, pharmacy, and food industries, among others. Rani et al. [22] focused on scarcely investigated, marine-microorganism-derived antioxidant substances: pneumocandins, taxol, astaxanthin, hispinin, and its derivates. Meanwhile, Begun et al. [35] presented antioxidant compounds derived from brown seaweeds: sulfated polysaccharides, polyphenols, carotenoids, and sterols. Ezquerra-Brauer et al. [94] compiled studies on establishing and elucidating the mechanisms of action of marine-species-extracted biochemical compounds with antioxidant activities, including pigment proteins from cyanobacteria, to determine their possible applications in various industries.

**Figure 5:**
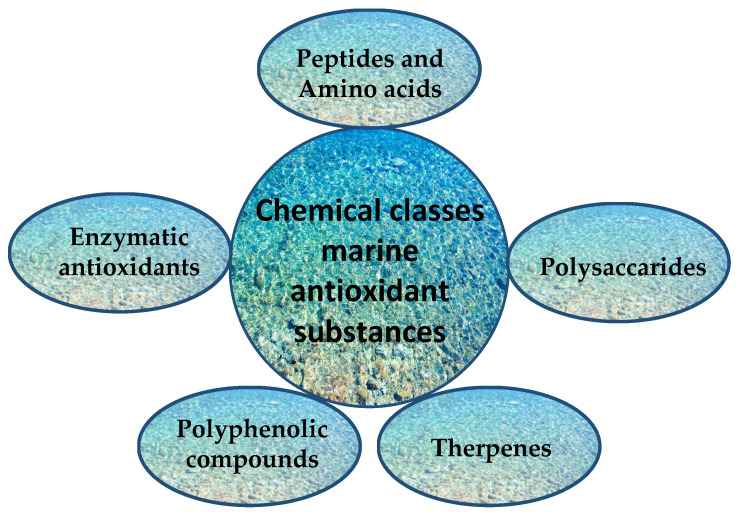
Main chemical classes of antioxidant substances derived from marine sources.

Here, we present antioxidant substances derived from marine sources during the last 5 years, grouped according to their chemical nature. They are summarized in Figure 5 and Supplementary Table S2.

The presentation includes known antioxidant substances derived from new sources and newly identified antioxidant compounds in known marine organisms, as well as a brief description of their characteristics, antioxidant activity, and potential applications. We include scarcely investigated new multifunctional peptides and those derived from marine byproducts and wastes, as well as antioxidant peptides that are highly active against alcohol toxicity; we also describe new scalarane-type sester-terpenes and sulfated polysaccharides from red algae. For the first time, we also reveal the effect of chito-oligosaccharides’ sequence on their antioxidant activity, etc.

### 4.1. Peptides and Amino Acids

#### 4.1.1. Antioxidant Peptides

Peptides are short or long chains of amino acids with different structures and molecular weights. The bioactive peptides usually have between 2 and 20 amino acid residues and demonstrate activity after being released from the main protein. Their antioxidant effect is one the most important, together with various other biological activities—antimicrobial, anticancer, antihypertensive, anti-inflammatory, etc.—and functional properties such as foaming, emulsifying, and solubility, which could be beneficial for industrial application [18,95]. Despite their very challenging screening, extraction, and purification by traditional chemical methods, numerous antioxidant peptides have already been identified and many studies demonstrate that they have a positive effect on human health and can be applied in the food and cosmetic industries [96]. The antioxidant activity depends on the peptide structure and amino acid sequence. It is usually attributed to free radical scavenging, lipid peroxidation inhibition, and metal ion chelating. Antioxidant peptides could be derived from various marine sources, including waste or byproducts [96,97], using different technologies for extraction, purification, and identification, as well as different methods for the evaluation of their antioxidant and other biological activities [98,99]. Their industrial-scale production is hampered by different problems, such as high production costs and low yield bioactivity. New microwave, high-pressure, pulsed electric field and other derivation technologies are reported to overcome the problems of the conventional hydrolysis methods—chemical, enzymatic, or by fermentation [100].

Chemical analysis as well as MS spectra of a fractionated and purified crude methanol extract obtained by freeze-thawing indicate that the antioxidant fraction derived from the marine bacteria *K. marina CDMP 10* contains short-chain peptides [72]. The antioxidant activity of three highly hydrophobic peptides, Ser–Ser–Gln, Phe–Glu, Asp–Ile, and Leu–Glu, was confirmed in vitro and by a cell-based assay. These small peptide molecules are non-cytotoxic and can protect human cells from chemical-induced oxidative stress. The Ser–Ser–Gln peptide demonstrates potential free radical scavenging activity in hepatocellular carcinoma cell lines, suggesting that it may serve as a potential pharmaceutical candidate with antioxidant activity [72]. Three classes of water-soluble phycobilin proteins, phycoerythrin, phycocyanin, and allophycocyanin, which constitute up to 60% of the total soluble cellular proteins in microalgae, are well known for their strong antioxidant and free radical scavenging activities [101]. Because of their high commercial value as natural colorants in the nutraceutical, cosmetic, and pharmaceutical industries, investigations to optimize their extraction continue [102].

Abuine et al. [103] found that short-chain peptides purified from fish skin hydrolysates demonstrate biological activities that are based on their amino acid composition and sequence. High content of hydrophobic amino acids contributes to the antioxidant and angiotensin-converting enzyme inhibitory activity [103]. The antioxidant peptide Leu-Trp–His–Thr–His (LWHTH), purified from the peptic hydrolysate of the edible marine animal *Styela clava*, has potential to be a healthy functional food with an antihypertensive effect, as was demonstrated by Kang et al. [104]. Cyanobacterial pigment phycobilin proteins (PBPs) are the major light-harvesting pigment proteins, with widely characterized in vitro and in vivo antioxidant activity [105]. Since reactive oxygen species (ROS) are considered an important factor in aging, PBPs can be used as effective free radical scavengers and are a potential candidate to develop anti-aging drugs. Sonary et al. [105] discussed other possible mechanisms behind the anti-aging activity of these ecologically and economically important biomolecules. Guo et al. [106] derived a peptide-rich protein hydrolysate from the sea cucumber *A. japonicas*. In vivo antioxidant capacity testing indicated that it is capable of increasing the survival rate and reducing the ROS level in an animal model scavenging DPPH free radicals. Enzymatic peptides from the swim bladder of Atlantic cod *Gadus morhua*, studied by Li et al. [107], demonstrate in vitro antioxidant activity (DPPH, ABTS, FRAP assays) and anti-aging properties. Quan et al. [108] reported antioxidant peptide fractions obtained by the optimized enzymatic hydrolysis of oyster soft tissue. Shaibani et al. [109] derived low-molecular-weight fractions with high content of hydrophobic amino acids (48.87% for 3–10 kDa; 46.26% for <3 kDa) bfrom protein hydrolysates of Rocky Shore Crab *Grapsus albacarinous*, using an ultrafiltration membrane. Their high antioxidant activity and remarkable cytotoxic effect against MCF-7 cancer cells were demonstrated.

##### Antioxidant Peptides from Byproducts and Wastes

In recent years, antioxidant peptides have been reported that are derived from marine byproducts and wastes. Pérez-Gálvez et al. [110] discussed fish discards as a source of health-promoting bio-peptides. Siera et al. [111] reported antioxidant peptides derived from enzymatic hydrolysates (by Alcalase^®^ 2.4 L) of red tilapia (*Oreochromis* sp.) ground scales, which are a byproduct of the aquaculture industry. The hydrolysate was fractionated to purify and identify the antioxidant peptides by membrane ultrafiltration and chromatography. The hydrophilic ultra-filtrated fraction with a molecular weight of 3–10 kDa and a sequence of twenty antioxidant peptides, containing 6–16 amino acids, was shown to have the highest antioxidant activity [111]. Usak et al. [112] reported the functional and bioactive properties of peptides derived from marine side streams in fish processing, including skin, bones, heads, and viscera. Such streams are rich in bioactive nitrogenous compounds and proteins, which can be converted into peptides through enzymatic hydrolysis or bacterial fermentation. These bioactive peptides can be used as modifiers of food ingredients’ solubility, water-holding and fat-binding capacity, and gelation, or to prevent food spoilage; they also may be used as antioxidants in the pharmaceutical industry, but also as antihypertensive, anticoagulant, and immunomodulatory compounds [112].

##### New Antioxidant Peptides

Chen et al. [113] discovered a new peptide that is highly active against alcohol toxicity in HepG2 cells. It is purified from enzymatic hydrolysates (using chymotrypsin, trypsin, pepsin, and in vitro gastrointestinal digestion) of brown-golden marine microalga *Isochrysis Zhanjiangensis.* The amino acid sequence and molecular mass of the purified peptide was identified as Asn–Asp–Ala–Glu–Tyr–Gly–Ile–Cys–Gly–Phe. Its protective effect was first investigated against ethanol-induced oxidative stress in HepG2 cells [113]. Bashir et al. [114] identified and characterized new antioxidant peptides from mackerel (*Scomber japonicus*) muscle protein hydrolysates that could be used as functional ingredients in the pharmaceutical industry. The peptide showing the highest DPPH scavenging activity was ALSTWTLQLGSTSFSASPM [114]. New multifunctional peptides are also presented in the literature, such as myofibrillar proteins derived from *Trachinus Draco* (greater weever), which have DPPH antioxidant and metal chelating activities [115]. Sun et al. [116] showed that peptides from yak (*Bos grunniens*) bone hydrolysates (papain and alcalase hydrolyzation) have strong antioxidant activity (DPPH, ABTS, FRAP assays and simulated gastrointestinal digestion in vitro) and potential as a new type of natural antioxidants. Among 10 peptides, three, namely Gly–Phe–Hyp–Gly–Ala–Asp–Gly–Val–Ala, Gly–Gly–Pro–Gln–Gly–Pro–Arg, and Gly–Ser–Gln–Gly–Ser–Gln–Gly–Pro–Ala, possess strong antioxidant activities. Gly–Phe–Hyp–Gly–Ala–Asp–Gly–Val–Ala has also a significant cytoprotective effect in Caco-2 cells under induced H_2_O_2_ oxidative stress. It reduces the formation of ROS and malondialdehyde, which improves the activity of antioxidant enzymes in the cells [116]. Antioxidant peptides from Atlantic red seaweed *Porphyra dioica Conchocelis* were, for the first time, derived by Pimentel et al. [117] using a specific combination of proteases (Prolyve^®^ and Flavourzyme^®^). The molecular mass distribution of the hydrolysate, the free amino acid content, and the antioxidant activity were determined by a range of in vitro assays. The significant improvement in the antioxidant activity of the hydrolysates compared to the control (up to 2.5-fold) indicates their potential as a novel source of antioxidant ingredients.

#### 4.1.2. Amino Acids

Amino acids are another type of substance with antioxidant activity. Mycosporine-like amino acids (MAAs) are ultraviolet (UV)-absorbable compounds, naturally produced by cyanobacteria and algae. Other marine organisms also utilize MAAs to protect their DNA from UV-induced damage. The content of MAAs in marine organisms depends on the environmental conditions and the season. Nishida et al. [118] derived MAAs from red alga *Dulse palmaria palmata* (Usujiri, Hokkaido, Japan) by water followed by methanol extraction. The antioxidant capacity of the crude extract, purified palythine, and porphyra-334 was determined by ABTS radical scavenging and FRAP assays under various pH values. The highest scavenging activity and reducing power were found under alkaline conditions (pH 8.0) [118]. Vega et al. [119] derived and analyzed MAAs from cyanobacteria and red alga. Extracted in different solvents (water, ethanol, and combination water:ethanol), MAAs were the main molecules with antioxidant and photoprotective capacity, together with scytonemin and phenolic compounds. Mycosporine glutaminol was, for the first time, found in *Scytonema* sp., as identified by the maximum MAA absorption in the UV band. The antioxidant activity of cyanobacterium extracts appears to be higher than that of red macroalgae [119]. A positive correlation of antioxidant activity with the amount of MAAs, polyphenols, and biliproteins was observed [120]. Small-molecule antioxidants in marine organisms from the Great Barrier Reef, Japan, and the USA were reported [121]: zoanthid *Palythoa tuberculosa* and the ascidian, *Lissoclinum*; coral trout (*Plectropomus leopardus*); *Porphyra tenera*; *Mastocarpus stelatus*. Methanol–aqueous extracts containing MAAs suggest that mycosporine glycine may function as a biological antioxidant in marine organisms [121].

### 4.2. Polysaccarides

Polysaccharides are composed of various linear or multi-branched monosaccharides. Multiple factors, including molecular weight, monosaccharide composition and structure, sulfate position, and sulfurization degree, are known as determining the antioxidant activity of marine polysaccharides. Derived from a variety of marine sources, structurally diverse and with specific properties, they could be of interest for novel therapies and industrial applications, including nutraceuticals, pharmaceuticals, and functional foods [17]. Zhong et al. [120] summarized the chemical composition, structural characteristics, and antioxidant capacity of known antioxidant polysaccharides, as well as their in vivo protective effects mediated by antioxidative stress. Khora and Navya [122] described the bioactivity features of polysaccharides from marine seaweeds, including their antioxidant, immune-stimulating, antiviral, anticancer, antibacterial and antifungal, anti-inflammatory, anti-allergic, and anticoagulant activity, etc. Studying polysaccharide fractions from ethanol and water extracts of 15 seaweeds (*Dictyota dichotoma var. velutricata*, *Dictyota indica*, *Iyengaria stellata*, *Padina pavonia*, *Sargassum swartzii*, *Sargassum variegatum*, *Stoechospermum marginatum*, *Stokeyia indica*, *Jolyna laminarioides*, *Caulerpa taxifolia*, *Halimeda tuna*, *Ulva fasciata*, *Ulva lactuca*, *Solieria robusta*, and *Melanothamnus afaqhusainii*), Tariq et al. [123] demonstrated concentration-dependent antioxidant activity as well as variations in the antioxidant potential, evaluated by different in vitro assays. El-Shafei et al. [17] demonstrated that the amount of sulfurized polysaccharides in metabolite extracts from three macroalgae classes—*Phaeophyceae*, *Rhodophyceae*, and *Chlorophyceae*—correlates with their antioxidant activity. Swaminathan et al. [124] reported the in vitro free radical scavenging potential (by DPPH, H_2_O_2_, NO, FRAP, ABTS assays) of the polysaccharide L-Fucose isolated from ethanol and acetone extracts of the brown macroalgae *Padina gymnosporat*.

Glycosaminoglycans (negatively charged polysaccharides) are known to affect the regeneration of the mammalian central nervous system. Data from Sousa et al. [125] suggest that the dermatan sulfate, obtained from the invertebrate ascidian *Phallusia nigra*, reduces ROS and has neuroprotective and antioxidant action even under neurodegenerative conditions caused by rotenone, i.e., this dermatan sulfate is responsible for the antioxidant activity and neuroprotection in the neuroblastoma-2A cell line.

Marine chito-oligosaccharides are known to have good antioxidant activity that is closely related to their sequences. To evaluate the specific structure–antioxidant activity relationship, Hao et al. [126] prepared chitosan dimers with different sequences and, for the first time, revealed the effect of chito-oligosaccharides’ sequences on their antioxidant activity. It was found that the amino group at the reducing end plays a vital role in the scavenging of superoxide radicals and in the reducing power of the chitosan dimer.

Sulfated polysaccharides from brown, green, and red algae—respectively, fucoidans, ulvans, and carrageenans—are reported to have antioxidant activity, despite their structural and nutritional features. The cell walls of the macroalgae are rich in sulfated polysaccharides, some of them being better nitric oxide scavengers than commercial antioxidants such as butylated hydroxyanisole [127]. The antioxidant activity of the sulfated polysaccharides depends on the type of algae from which they are derived, as well as on their chemical structure, the degree of sulfation and molecular weight, the type of the major sugar, and the glycosidic branching. It is known that low-molecular-weight sulfated polysaccharides have higher antioxidant activity because they may be incorporated into the cells, and they can donate protons more effectively than high-molecular-weight sulfated polysaccharides [17]. Jose and Kurup [128] reported the superior antioxidant activity of sulfated polysaccharides, isolated from the edible marine algae *Sargassum swartzii*, from the Kerala coast, India, that are suitable for antioxidant therapies.

#### 4.2.1. Polysaccharides from Brown Algae

Derived from brown algae, sulfated polysaccharides attract extensive research interest due to their numerous biological activities. Guru et al. [129] demonstrate the high antioxidant activity and free radical scavenging capacity (ABTS, DPPH, FRAP assays, and TAP) of sulfated polysaccharides from the hot water crude extract of brown algae *Turbinaria ornate*. Two concentrated fucoidan-rich extracts from marine macroalga *Undaria pinnatifida* (85% fucoidan) and *Fucus vesiculosus* (co-extract, 60% fucoidan, 30% polyphenol) were demonstrated to have topical benefits in comparative in vitro and in double-blind, placebo-controlled clinical studies [130]. The major effects of the *U. pinnatifida* extract are the aiding of skin immunity, soothing, and protection, while the *F. vesiculosus* extract most significantly affects age spot reduction and increases brightness, soothing, and protection [130]. Liu et al. [131] derived four fucoidan fractions from water extracts of brown seaweed *Sargassum pallidum* (Yellow Sea, China). These fractions were found to consist of fucose, rhamnose, xylose, mannose, glucose, and galactose, with different monosaccharide molar ratios. They have potential to be used as a pharmaceutical resource and functional food [131]. Kordjazi et al. [40] found that fucoidans extracted from two brown seaweeds, *Sargassum ilicifolium* and *Sargassum angustifolium* (Qeshm Island, Iran), have chemical compositions and antioxidant and antimicrobial properties indicating their possible application in the nutraceutical industry. Jayawardena et al. [132] studied the protective effect against oxidative stress—in vitro and in vivo in a zebrafish model—of sulfated polysaccharides (majority fucoidans) from the celluclast enzyme-assisted extraction and ethanol precipitation of brown algae *Padina boryana* (Maldives). The results suggest that they might be potent, water-soluble, natural antioxidants for sustainable industrial applications. Laeliocattleya et al. [41] discussed the fucoidan content of the brown seaweed *Sargassum filipendula* and its free radical scavenging potential. The latter depends on the composition of the majority of fucoidan-sulfated polysaccharides and small amounts of monosaccharides such as galactose, xylose, glucose, and mannose. However, the content and activity of these compounds are influenced by the extraction method, temperature, time, and solvent concentration.

#### 4.2.2. Polysaccharides from Green Algae

Green algae are rich in ulvan (*Ulva* species), sulfated rhamnan (*Monostroma species*), and galactan (*Codium species*), with main applications in agriculture and less in pharmacy [133,134,135,136]. Ulvan is composed mainly of mono- or disaccharide units such as sulfated rhamnose, sulfated xylose, and uronic acids (glucuronic acid and iduronic acid), whereas the remaining sulfated polysaccharides in the *Ulva* species are cellulose, xyloglucan, and glucuronan [17]. The composition of ulvan varies with the source of the species, the storage conditions of the collected biomass, pre-extraction processing, extraction method, and the processing procedure. The low water solubility of ulvan, its interactions with cell wall components (divalent cations, e.g., calcium ion), hydrogen bonding, and other physicochemical properties determine the selection of the extraction method and operation conditions [17].

#### 4.2.3. Polysaccharides from Red Algae

The sulfated polysaccharide α-Carrageenan from red algae, with known antioxidant and free radical scavenging activity [137], was described in 2009 by Grassauer and Prieschl-Grassauer [138] to be able to facilitate protection from the newly discovered coronavirus, COVID-19, or at least to be used as a coating for protective masks and gloves. In 2020, Zaporozhets et al. [139] confirmed the earlier claims of Grassauer and Prieschl-Grassauer, demonstrating the significant anti-coronavirus activity of sulfated polysaccharides extracted from *S. japonica*, one of the most widely consumed seaweeds in China and Japan. Alencar et al. [140] isolated and determined the chemical structure of new sulfated polysaccharides from the enzymatic extract of the red algae *Gracilaria caudate* (SP-Gc) and demonstrated that SP-Gc may be used as a hydrocolloid. Its significant, concentration-dependent, in vitro antioxidant activity was proven. Its in vivo antioxidant activity was demonstrated by CAT and SOD level measurement, in an 2,2′-azobis(2-methylpropionamidine)dihydrochloride (ABAP)-induced oxidative stress model, of rats pre-treated with SP-Gc (3 and 10 mg/kg) [140]. Khan et al. [141] found that the polysaccharides derived from the alga *Porphyra haitanensis* primarily contain galactose and 3,6-anhydrogalactose in a molar ratio of 1.2:1.0, respectively. They revealed its relatively high ABTS radical scavenging activity, moderate DPPH radical scavenging efficacy (34.63% at 2 mg/mL), and low hydroxyl radical scavenging potential (23.80% at 2 mg/mL).

### 4.3. Terpenes

**Figure 6:**
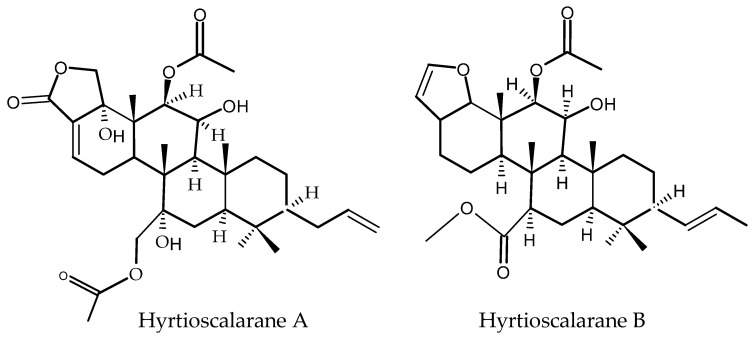
Chemical structure of hyrtioscalaranes A and B, isolated from the demosponge *Hyrtios erectus*.

Terpenes, formed by the condensation of two subunits of isoprene (C_5_H_8_), known also as terpenoids, are antioxidant molecules with a very diverse structure [18]. Two new scalarane-type sester-terpenes, hyrtioscalaranes A and B (see their chemical structure in Figure 6), were isolated by K Chakraborty and Francis [142] from the organic extract of the demosponge *Hyrtios erectus* through extensive chromatographic purification.

The evaluation of their antioxidant and anti-inflammatory effects showed that hyrtioscalarane A exhibits greater antioxidant activity (ABTS, DPPH free radical quenching) than hyrtioscalarane B and the antioxidant α-tocopherol. Both hyrtioscalaranes A and B display a higher selectivity index (>1) than the commercial anti-inflammatory agent ibuprofen (0.43) [142]. The antioxidant potential of the monoterpenoid (−)-Loliolide, isolated from seaweed *Sargassum horneri*, was revealed by Kim et al. [143] in Vero cells and in zebrafish models. Electron spin resonance demonstrated that (−)-Loliolide has significant alkyl radical scavenging activity (IC_50_: 0.043 ± 0.005 mg mL^−1^). Dose-dependent protective effects were found for Vero cells’ viability against 2,2′-azobis(2-amidinopropane)dihydrochloride (AAPH)-induced intracellular ROS and reduced lipid peroxidation in AAPH-induced zebrafish embryos [143]. Thyrsiferol, isolated from red algae (genus *Laurencia*), was found to show potential antiviral and antitumor activities, whereas terpenoids, derived from marine sponges (genus: *Insignia*), act as anti-inflammatory agents, analgesics, and antibiotics [144].

A variety of marine pigments are represented now as promising alternatives to the antioxidants and synthetic additives used today [91]. Carotenoids are the largest group of natural pigments responsible for the wonderful colors of marine organisms, demonstrating also antioxidant activity. The carotenoids are tetraterpenes, divided into two chemical classes: carotenes that are composed of hydrogen and carbon (such as lycopene, α- and β-carotene) and xanthophylls, which are constituted by hydrogen, carbon, and oxygen (such as astaxanthin, fucoxanthin, and lutein). The carotenoids are fat-soluble substances, biosynthesized by all autotrophic marine organisms, such as bacteria and archaea, algae, and fungi, but also by some others. The carotenoids perform functions in different ways and at different levels: (i) they act as quenchers of singlet molecular oxygen; (ii) they convert hydroperoxides into more stable compounds; (iii) they prevent the formation of free radicals through the blockage of free radical oxidation reactions and inhibition of auto-oxidation chain reactions; (iv) they convert iron and copper derivatives into harmless molecules, acting as metal chelators. Carotenoids are used mainly in the food, cosmetic, and pharmaceutical industries. In addition to their utilization for pigmentation, they have significant therapeutic applications, such as improving the immune system and preventing neurodegenerative diseases. The biotechnological production of natural carotenoids from marine organisms has considerably increased due to several advantages, such as costs, times, and yields, when compared to terrestrial plants or synthetic products [8,145]. The exogenous antioxidants α- and β-carotene, lutein, and astaxanthin, play important roles in the prevention of oxidative damage by free radical scavenging, protecting the lipid bilayer from peroxidation. The basis of this effect, as well as of the around 10-fold greater antioxidant activity of astaxanthin than that of other carotenoids (lutein, canthaxanthin, and β-carotene), is its unique molecular structure, characterized by polar ionic rings and non-polar conjugated carbon–carbon bonds [26]. Other antioxidant carotenoids are fucoxanthin, found in brown seaweeds and tunicates; shellfish mytiloxanthin, which is a metabolite of fucoxanthin; zeaxanthin, with antioxidant properties similar to those of the α-tocopherol; saproxanthin and myxol, two rare marine monocyclic carotenoids, isolated from bacteria from the family *Flavobacteriaceae*, which show antioxidant activity stronger than that of zeaxanthin and β-carotene [146]. Similar to other bioactive marine derivatives, the carotenoids (astaxanthin, fucoxanthin, β-carotene, lutein, and the rare saproxanthin, sioxanthin, and myxol) have low stability, are poorly absorbed, and have very limited bioavailability [146]. Nanoencapsulation is discussed as a tool to preserve the carotenoids and their original properties during processing and storage, to improve the physiochemical properties, and to increase the health-promoting effects [147].

Vasanthabharathi and Jayalakshmi [148] underline the importance of the brown-black pigment melanin from marine *Actinomycetes*, which are accepted as some of the novel producers of bioactive molecules. It was shown that melanin protects microorganisms against UV radiation, enzymatic lysis, oxidation, and killing by alveolar macrophages. The understanding of the importance and novelty of melanin from marine actinomycetes is very low.

### 4.4. Polyphenolic Compounds

Polyphenolic compounds are secondary metabolites produced in marine organisms as a response to different stress conditions. Similar to natural peptides, MAAs, polysaccharides, and terpenes, polyphenols exhibit antioxidant activity. Based on their main structure, polyphenols are classified into several groups: flavonoids, phenolic acids, stilbenes, and lignans [18]. Fernando et al. [149] summarized the knowledge about antioxidant polyphenolic compounds in marine algae in an attempt to describe the structure–antioxidant activity relationship. Mateos et al. [150] presented examples of major types of antioxidant phenolic compounds, divided into three families: (i) bromophenols; (ii) simple phenolic acids and flavonoids; and (iii) phlorotannins (fucols, phlorethols, fucophlorethols, fuhalols, carmalols, and eckols). These phenolic compounds can act as efficient antioxidants through different mechanisms: singlet oxygen and free radical scavengers, reducing or/and chelating agents, etc. [150]. Jimenez-Lopez et al. [151] presented the main bioactive phenolic compounds in marine algae, comparing the effectiveness of different techniques for their extraction as well as available methods for their identification and quantification; they also described the stability of extracts enriched in phenolic compounds and the main bioactivities of the secondary metabolites.

Ovothiols are thiol histidine derivatives with unusual antioxidant properties and anti-proliferative and anti-fibrotic activities due to the position of the thiol group on the imidazole ring of the histidine [152]. Ovothiols are synthesized by two enzymes: sulfoxide synthase OvoA and sulfoxide lyase Ovo B. Three differentially methylated forms (A, B, and C) of ovothiols could be isolated from the ovaries, eggs, and biological fluids of marine pathogenic protozoa, microalgae, invertebrates, and mollusks [152].

Synthetic phenolic antioxidants (SPAs) are widely used in various industrial and commercial products to retard oxidative reactions and to lengthen the lifetimes of products, but there are a number of toxicity studies suggesting that some SPAs and their transformation products may cause non-desirable effects for humans and the environment, which makes the natural polyphenolic antioxidants preferable in this respect [152].

### 4.5. Enzymatic Antioxidants

The enzymatic inhibitors are an important group of compounds among the biologically active substances, derived from marine organisms and their metabolites. New discoveries of these antioxidants are expected from the use of novel derivation and characterization techniques [153]. Qiao et al. [154] presented the molecular characterization, purification, and antioxidant activity of recombinant superoxide dismutase (SOD) enzyme from the Pacific abalone *Haliotis discus hannai Ino* (*Pichia pastoris*) (HdhCu/Zn-SOD). For the first time, they reported the heterologous expression of HdhCu/Zn-SOD in *P. pastoris*, the antioxidant activity of this acidic metallic enzyme, and its action as a scavenger of endogenously and exogenously produced free radicals [154]. Espinosa-Ruíz and Esteban [155] investigated wound-induced changes in antioxidant enzyme activities in skin mucus and gene expression in the skin of Gilthead Seabream (*Sparus aurata* L.). This is the first work on the determination of the effects on a wound in different skin areas of one fish. [155]. A new digestive α-amylase from Blue Crab (*Portunus segnis*, *P. segnis*) viscera was reported to be purified, biochemically characterized, and applied for the improvement of the antioxidant potential of oat flour [156]. The enzyme (molecular weight of approximately 45 kDa) was purified by ultrafiltration and Sepharose mono Q anion exchange chromatography. The α-amylase could hydrolyze carbohydrates, producing maltose, maltotriose, and maltotetraose as the major end products of starch hydrolysis. The α-amylase from *P. segnis* provides novel features compared with other marine-derived enzymes and a better understanding of the carbohydrate biodegradability in marine environments, particularly in invasive alien crustaceans [156].

## 5. Marine Antioxidant Derivation Technologies

The biotechnological production of antioxidants from marine biota starts with the preparation of crude extracts (total or sequential) and the evaluation of their biological activity, followed by the purification and identification of antioxidant substances and, in rare cases, the development of synthetic copies of corresponding natural antioxidants.

### 5.1. Conventional Solid–Liquid Extraction

Solid–liquid extraction is the conventional process to obtain antioxidant substances or extracts. It includes several stages: pre-treatment of the row biomass, such as drying, grinding, swelling, etc., to improve the penetration of the solvent/solvent system; total or sequential extraction with a relevant solvent/solvent system to derive the desired crude extract or antioxidant substances; post-treatment of the liquid extract, including concentration, purification, filtration, etc.; removal of the solvent, and, usually, drying [157,158]. The solid–liquid extraction depends on several factors, such as the applied extraction technique, operation conditions (temperature, time, pH, solvent, etc.), and composition of the raw material [45,159,160].

Among the various extraction factors, solvents play an important role in the efficiency of the extraction. A suitable solvent can derive safe and high-quality ingredients and preserve the biological effects of the extracted compounds, and it is recyclable and reusable, preventing negative environmental effects. There is a wide range of solvents with different polarities, which allows the selective extraction of the target family of compounds [161]. The selection of the solvent is based on the chemical nature and polarity of the compounds to be extracted—compounds with different chemical structures require solvents with different polarities [162]. For example, carotenoids are lipid-soluble antioxidants, and organic solvent systems, such as mixtures of hexane with acetone, ethanol, or methanol, or mixtures of ethyl acetate with acetone, ethanol, or methanol, are used for their extraction [163,164]. Most of the phenolic antioxidant compounds are hydro-soluble. Therefore, polar and medium-polar solvents (water, ethanol, methanol, propanol, acetone, and their aqueous mixtures) are used for the extraction of phenols [165]. For example, four fractions of water-soluble fucoidans with different molecular weights were extracted from brown seaweed using cold and hot water extraction, and fractional precipitation with gradient concentrations of ethanol [131]. The solvent influences the antioxidant effects of the extracts due to the derivation of different antioxidant compounds by different solvents [166]. Studying the effect of four solvent systems on the phenolic content and antioxidant activity of extracts from selected macro- and microalgae, Monteiro et al. [158] found that the solvent system influences the composition and biological activity of the extracts. A lower organic solvent:water ratio increases the efficiency of macroalgae biomass extraction but not that of microalgae. Total phenol, ortho-diphenol, and flavonoid amounts are highly influenced by the used solvent system and algae material. Microalgal extracts with trong ABTS scavenging activity can be obtained with ethanol:water = 80:20 (*v*/*v*), while no visible trend has been detected for macroalgae extracts [158]. Methanol extracts are the most effective in the scavenging of DPPH; a positive correlation was observed between phenolic content and antioxidant capacity for macroalgae extracts, while the opposite was observed for microalgal extracts, suggesting that, in microalgae, phenolic compounds are not the main contributors to the observed antioxidant capacity [158]. The effects of the extraction solvents on yet unexplored bioactive compounds from marine seaweeds and their antioxidant activity were observed also by Sobuj et al. [45].

The conventional solid–liquid extraction process has a number of drawbacks, such as the use of huge amounts of often harmful (toxic and/or flammable) solvents; the presence of residual solvents and contaminants from the raw material in the extracts; the presence of denatured compounds due to extreme extraction conditions; long treatment times; the consumption of large amounts of energy; and the generation of large amounts of waste [162,167]. Experiments to address some of these issues has been carried out. For example, to improve the conventional extraction of bioactive compounds from marine cyanobacteria, Assunção et al. [86] used a combined safe solvent extraction, enhanced by preliminary treatment with sulfuric acid, NaOH, PBS, DMSO 20%, DMSO 100%, or acetone. The pre-treatment with PBS appeared to be the most effective.

An optimized process is expected to reduce the extraction time and energy consumption, the solvent amount and environmental impact, the economic costs, and the waste quantity, as well as to ensure high-quality and safe extracts [157,168]. A number of improved alternative technologies to the conventional technique (Soxlet, heat reflux, infusion, distillation, etc.) have been proposed to extract target antioxidant compounds from marine biota.

### 5.2. Non-Conventional Derivation Technologies

To mitigate the drawbacks of conventional solid–liquid extraction, newly emerging technologies are receiving huge interest around the world [18,165], such as enzyme-assisted (EAE) [165,169], microwave-assisted (MAE) [165], or ultrasound-assisted extraction (UAE) [52,61,165,170]; pressurized liquid (PLE) [165], high hydrostatic pressure (HHP) [165], and supercritical fluid extraction (SFE) [165]; pulsed electric field (PEF) [171], high voltage electrical discharge (HVED) [171], and ohmic heating (OH) extraction [171], etc. Santos et al. [18] discussed different non-conventional extraction strategies as sustainable alternative techniques to preserve the potency of the antioxidants and antiviral compounds extracted from marine sources. Jin et al. [169] presented recent advances in the extraction of peptides from marine organisms and the biological activity of various marine-organism-derived peptides.

Getachew et al. [165] focused on emerging technologies for marine phenolic substances’ extraction, such as enzyme-assisted extraction (EAE), microwave-assisted extraction (MAE), ultrasound-assisted extraction (UAE), pressurized liquid extraction (PLE), and supercritical fluid extraction (SFE).

To avoid the degradation of carrageenans from *Hypnea musciformis* (collected from Saint Martin Island, Bay of Bengal, Bangladesh), Rafiquzzaman et al. [170] used the rapid UAE of aqueous- and alkali-treated biomass. Ding et al. [52] prepared an ultrasonic extract of red sea cucumber, *S. japonicus* (collected from Jeju Island), that showed significant antioxidant and anti-melanogenesis activities. For the first time, Chzhu et al. [61] extracted complexes of biologically active substances from Black Sea invertebrates by the resource-light method of two-phase extraction in combination with ultrasonication and proposed conditions for obtaining extracts with optimal characteristics.

Yuan and Macquarrie [172] used microwave extraction to derive sulfated polysaccharides (Fucoidan) from the brown seaweed *Ascophyllum nodosum*. The highest antioxidant activity was found for fucoidan extracted at 90 °C. Pierucci et al. [173] explored microwave extraction as a tool to improve the fucoidan yield from *Fucus* brown algae. The extracts were purified by dialysis. The results suggest that, in order to maximize the fucoidan yield, one should tailor the extraction method to the specific algae species.

Electro-technologies, based on the direct application of an external electric field through a semi-conductive material, are one method among a wide range of biotechnological processes considered cost-effective and environmentally friendly in view of the less intensive use of non-renewable resources and high levels of energy efficiency. Recent fundamental research gives reason to expect that pulsed electric fields (PEF) and moderate electric fields (MEF), targeted at microalgae cellular permeabilization and the subsequent extraction of valuable compounds, will become mainstream techniques that, in the near future, could be applied to the industrial exploitation of microalgae [171]. Simulated human-like gastrointestinal digestion was proposed recently by Borawska-Dziadkiewicz et al. [174] for the release of peptides from salmon and carp proteins. The new technologies have the advantage of reduced extraction time and solvent quantity in comparison to the traditional methods [52,61,165,169,170,171].

This short presentation of the current conventional solid–liquid extraction and non-conventional derivation methods demonstrates that the new, non-conventional ones avoid some drawbacks of conventional extraction, reducing the extraction time, solvent, and waste quantity; increasing the yield of the target substances, and preserving their biological activity; thus, the new technologies provide sustainable alternatives to preserve the potency of antioxidant substances and other biologically active compounds extracted from marine biota.

## 6. Concluding Remarks

The interest in high-value antioxidant substances from marine sources has continued during the last few years. Due to the sharply increasing microbial resistance to antibiotic and multidrug treatments, natural substances combining antioxidant and antimicrobial activities in one molecule are of particular interest.

New marine sources of natural antioxidants, novel bacterial strains, new antioxidant metabolites, new antioxidant activities, and their pharmaceutical and other applications have been identified. New substances with antioxidant activity are reported, along with those extracted from marine organisms and metabolites collected from other locations, since their biological activity depends on the climatic conditions under which marine organisms live. Along with a variety of marine biota, byproducts and wastes offer valuable sources of substances with antioxidant properties.

The use of new derivation approaches and optimized extraction processes allows the intensification of the production and improvement of the quality of the target substances. The non-conventional derivation technologies, such as microwave-assisted, ultrasound-assisted, or subcritical water extraction and others, surpass the conventional methods in terms of extraction efficiency, the potency of the active substance, as well as environmental preservation. Therefore, future research should be focused on their implementation in practical applications and the assessment of the potential issues.

It is difficult to state which organisms or extracts/compounds are the most promising due to the importance not only of their content but also of the specific complex of properties relevant to different applications, as well as the influence of the derivation biotechnology and the operation conditions.

Knowledge about the biological effects and pharmacological properties of new antioxidant compounds would provide data for their practical application in the treatment of human disorders, infectious diseases, antibiotic resistance, cancer, and many others. The ability to obtain safe, high-value products is of key importance for future potential industrialization.

## Supplementary Materials

The following supporting information can be downloaded at: https://www.mdpi.com/article/10.3390/antiox11030439/s1, Table S1: Marine sources of natural substances with antioxidant activity; Table S2: Substances with antioxidant activity derived from marine biota.
