# Supplementary material for: Recent Progress in Antioxidant Active Substances from Marine Biota
Source: Antioxidants (Basel). 2022 Feb 22;11(3):439. doi: 10.3390/antiox11030439 (PMC8944465; doi:10.3390/antiox11030439)
Supplement: Supplementary file 1 [file antioxidants-11-00439-s001.zip › Last_Table S1.pdf]

Table S1. Marine sources of natural substances with antioxidant activity

Source	Location of harvesting	Extracts Hydrolysates Biomass	Biological activity Potential applications	Ref.
**Marine macro-organisms**				
**Seaweeds**				
brown seaweeds				
Sargassum ilicifolium, Sargassum angustifolium, Sargassum filipendula	Qeshm, Island, Iran Indonesia	Fucoidan extracts	Antioxidant Antimicrobial Nutraceutical industry	[40] [41]
Turbinaria decurrens Bory	Fillipins	Fucoidan extracts	Radicals scavenger Cosmetics Drug-resistant bacterial infections Diabetes	[42]
Toania atomaria (Phaeophyta)	Rocky Bay, Abu Qir, Alexandria, Egypt	Ethanol extract	High antioxidant	[43]
Caulerpa racemosa var. cylindracea	Algeria coast	Crude extracts containing new bioactive components	Antioxidant Antibacterial	[44]
Unexplored *Padina tetrastromatica, Gracilaria tenuistipitata*	Bangladesh	Various solvent extracts	Antioxidant	[45]
Zonaria tournefortii	-	Acilphloroglucinol derivatives	Antioxidant	[46]
Ecklonia maxima blades<br>*Ecklonia maxima* stipe	-	Hydrolysates (celluclast-assisted; viscozyme-assisted)	Antioxidant Anti-inflammatory Functional food	[47]
Caulerpa racemose	-	Maceration extracts	Very strong antioxidant UV protector	[29]
Red seaweeds				
Gracilaria bursapastoris	Nador laoon, Maroco	Extracts (ethanol)	Antioxidant	[48]
Asparagopsis armata, Gracilaria gracilis, Hypnea musciformis, Laurencia obtusa, Pterocladiella capillacea, Sphaerococcus cornopifolius	north coast of Tunisia	Extracts (methanol)	Radical scavenging	[49]
**Sea cucumbers**				
Holothuria tubulosa Gmelin 1791	Turkey seas	Extracts (acetonitrile/trifluoroacetic acid, methanol, water/methanol)	Antioxidant	[50]
Holothuria tubulosa tegument	Bizerta	Extracts (chloroform-methanol mixture)	Antioxidant	[51]

Source	Location of harvesting	Extracts<br>Hydrolysates<br>Biomass	Biological activity<br>Potential applications	Ref.
	lagoon, northern Tunisia			
Stichopus japonicas<br>*(S. japonicas)*	Red sea	Ultrasonic extracts (water)	Antioxidant<br>Anti-melanogenic	[52]
Holothuria leucospilota	Indonesian sea	Methanol extracts	Highest Antioxidant (among 16 from genus *Actinopyga, Bohadscia, Holothuria, Pseudocolochirus*, and *Stichopus)*	[53]
Holothuria atra	Indonesian sea	Extracts (methanol)	Highest antioxidant (among 21 other cucumbers from Indonesian sea)	[54]
S. japonicas	-	Hydrolysates<br>Enzymatic (alcalase, α-chymotrypsin, flavourzyme, kojizyme, neutrase, papain, pepsin, trypsin protamex)	Antioxidant (against H_2O_2-indused oxidative stress)	[55]
**Fishes**				
Raja porosa skate cartilage	-	Chondroitin sulphate (CS) extracted	Antioxidant (free radical scavenging more effective than shark CS)	[56]
Sardina pilchardus roe	-	Delivered lipids	Antioxidant<br>Anti-inflammatory (lipid liposomes)	[58]
**Marine invertebrates**				
**Marine sponges**				
Hyrtios erectus	North Bay, South Andaman Sea	Methanol extracts	Antioxidant<br>Anti-inflammatory	[62]
genus *Suberea*,<br>family *Aplysinellidae*	-	Produced bromotyrosine derivatives	Biological<br>Pharmacological	[63]
Diacarnus ardoukobae associated *Streptomyces sp. NMF6* strain	-	Ethyl acetate extract	Antioxidant<br>Anticancer<br>Antimicrobial<br>Antiviral	[64]
**Soft corals**				
Junceella junce Cavernularia sp.<br>*white Menella sp.*<br>*brown Menella sp.*<br>*Virgularia sp.*<br>*Sinularia compressa*<br>*Sinularia variablis*	Persian Gulf	Maceration extracts (methanol-ethyl Acetate)	Antioxidant<br>Anticancer	[65]

Source	Location of harvesting	Extracts Hydrolysates Biomass	Biological activity Potential applications	Ref.
Sinularia polydactyla				
Soft corals	-	Derived Sinularin, Dihydrosinularin	Antioxidant Anti-cancer	[66]
**Crabs**				
Grapsus albolineathus	rocky shore	Hydrolysate (Alcalase) (bioactive peptides)	Antioxidant Antibacterial	[67]
**Molluscs**				
Tympanotonus fuscatus var radula (Linnaeus) *Pachymelania aurita* (Muller)	NigerDelta, Nigeria	-	Antioxidant	[70]
jellyfish *Acromitus flagellatus*	Indian ocean	Nematocysts crude venom	Antioxidant (proteins, lipids, carote-noids)	[71]
**Microorganisms**				
**Microalgae**				
Dunaliella salina *Tetraselmis chuii* *Isochrysis galbana*	clone Tahiti	Extracts (methanol)	Antioxidant	[74]
Nannochloropsis oculata	-	Extracts (methanol)	Antioxidant Antimicrobial Anticancer	[75]
Nannochloropsis gaditana	-	Extracts (methanol)	Antioxidant Anti-inflammatory (in diabetic rats)	[76]
Galdieria sulphuraria, Ettlia carotinosa, Neochloris texensis, Chlorella minutissima, Stichococcus bacillaris, Schizochytrium limacinum, Crypthecodinium cohnii, Chlorella vulgaris	-	Crude extracts	Antioxidant Cytotoxic Therapeutic	[77]
Ninetyone (91) microalgae strains photo-tropically grown to generate biomass marine heterokonts *Bacillariophyte cf. Stauroneis sp. LACW24, Ocrophyte cf. Phaeothamnion sp. LACW34*	Irish waters	Extracts (polar solvents)	Biomass generation Antioxidant	[79]
Chlorella sp. S14	-	Biomass Polyunsaturated fatty acids (PUFA) extracts	Antioxidant Anti-proliferative	[80]
**Bacteria**				
New Gram-positive marine *Actinobacteria* strain	Unexplored sea sediment Alang Gulf,	Extracts (ethyl acetate)	Antioxidant Antibacterial	[82]

Source	Location of harvesting	Extracts Hydrolysates Biomass	Biological activity Potential applications	Ref.
	Khambhat, Gujarat			
Streptomyces sp. SCS525	Marine sediments	Extracts (ethyl acetate)	Strong antioxidant	[83]
Algae associated bacteria	Red Sea, Jeddah, Pakistan	Extracts (ethyl acetate) Secondary metabolites	Pharmac. industry (steroids, saponins, tannins, flavonoids, anthocyanin, betacyanin)	[84]
Bacillus<br>*Planococcus*	Saline Aushazia Lake, Saudi Arabia	Saline environment	Antioxidant (salinity stress enhanced)<br>Foods<br>Postbiotics	[85]
Cyanobacteria Chroococcidiopsis sp. LEGE 06174	-	Extracts (ethanol-PBS; acetone-PBS; methanol-PBS)	Antioxidant<br>Antioxidant industry	[86]
**Fungi**				
Aspergillus puulaauensis TM124-S4	-	Extract (water)	Skin protection	[90]
Sponge-associated endophytic fungi:<br>*Tedania anhelans*<br>*Myxilla arenaria*<br>*Callyspongia fibrosa*	east and west coasts of India	Sabouraud Dextrose Agar Medium	Antioxidant<br>Anticancer<br>Anti-inflammatory	[91]
Myrothecium sp. Bzo-l062	Deep sea	Extract (ethyl acetate)<br>Four new components derived	Antioxidant<br>Anti-inflammatory	[93]
