# Supplementary material for: Recent Progress in Antioxidant Active Substances from Marine Biota
Source: Antioxidants (Basel). 2022 Feb 22;11(3):439. doi: 10.3390/antiox11030439 (PMC8944465; doi:10.3390/antiox11030439)
Supplement: Supplementary file 1 [file antioxidants-11-00439-s001.zip › Last_Table S2.pdf]

Table S2. Substances with antioxidant activity derived from marine biota

Substances with antioxidant activity	Origin	Biological activity<br>Potential applications	Ref.
**Antioxidant peptides**			
Short chain hydrophobic peptides	Fish skin<br>Hydrolysates	Different<br>(dependant on the amino-acid composition and sequence)	[103]
Leu-Trp-His-Thr-His (LWHTH)	*Styela clava*<br>Peptic hydrolysate	Antihypertensive<br>Functional food	[104]
Peptide-reach protein hydrolisate	Sea cucumber, *A. japonicas*<br>Peptic hydrolysate	Reducing reactive oxygen species (ROS)<br>animal model	[106]
Swim bladder peptides	Atlantic cod<br>(*Gadus morhua*)	Antioxidant<br>Anti-aging	[107]
Peptide fractions	Oyster soft tissue<br>Enzymatic hydrolysate	Antioxidant<br>Anti-inflamatory	[108]
Low molecular peptide fractions	Rocky Shore Crab<br>(*Grapsus albacarinous*)<br>Protein hydrolysate	Antioxidant<br>Anti-cancer	[109]
From byproducts and wastes			
Biopeptides	Fish discards	Health promotion<br>Alternative foods	[110]
Hydrophilic peptide fraction<br>(molecular weight of 3–10 kDa, sequence of twenty peptides, containing 6–16 amino acids)	Red tilapia<br>(*Oreochromis sp*.)<br>ground scales<br>Enzymatic hydrolysates	Antioxidant	[111]
Bioactive peptides	Fish skin, bones, heads, viscera<br>Enzymatic hydrolysates	Antioxidant<br>Antihypertensive<br>Anticoagulant<br>Immunomodulatory<br>Anti-spoilage<br>Modifying solubility<br>Water-holding,<br>Gelation<br>Pharmaceutical ind.<br>Food	[112]
New discovered peptides			
Asn-Asp-Ala-Glu-Tyr-Gly-Ile-Cys-Gly-Phe.	Brown-golden marine microalga<br>*Isochrysis Zhanjiangensis*<br>Enzymatic hydrolysates<br>(chymotrypsin, trypsin, pepsin) *in vitro* gastrointestinal digestion	Anti-alcohol<br>in HepG2 cells	[113]
ALSTWTLQLGSTSFSASPM	Mackerel<br>(*Scomber japonicus)*<br>muscle<br>Protein hydrolysates	Antioxidant<br>Pharmaceutical industry<br>(functional ingredients)	[114]
Multifunctional peptides	*Trachinus Draco*<br>myofibrillar proteins<br>Protein hydrolisates	Antioxidant<br>Metal chelating	[115]

Substances with antioxidant activity	Origin	Biological activity<br>Potential applications	Ref.
Gly-Phe-Hyp-Gly-Ala-Asp-Gly-Val-Ala,<br>Gly-Gly-Pro-Gln-Gly-Pro-Arg,<br>Gly-Ser-Gln-Gly-Ser-Gln-Gly-Pro-Ala	Yak (*Bos grunniens*) bone<br>Papain and alcalase hydrolysates	Strong antioxidant	[116]
Antioxidant peptides	Atlantic red seaweed<br>*Porphyra dioica Conchocelis*<br>Protease hydrolysates<br>(Prolyve® and Fla-vourzym combination)	Antioxidant<br>(2.5-fold improved)	[117]
Amino-acids			
Mycosporine-like amino acids (MAAs)	Cyanobacteria<br>and red alga	Antioxidant<br>Photo-protective<br>Cosmeceutical	[119]
MAAs<br>Palythine<br>Porphyra-334	Red alga<br>*Dulse palmaria palmata*<br>(Usujiri, Hokkaido, Japan)<br>Water/methanol extracts	Antioxidant<br>Radicals scavenging	[118]
Polysaccarides			
L-Fucose	Brown macro-algae<br>*Padina gymnospora*<br>Extracts<br>(ethanol, acetone)	High free radical scavenging	[124]
Dermatan sulphate	Ascidian<br>*Phallusia nigra*	Antioxidant<br>Neuroprotection<br>Anti-cancer	[125]
Chitosan dimers<br>(with different sequences)	-	**For the first** time revealed sequence effect on antioxidant activity	[126]
Sulphated polysaccharides	Brown algae<br>*Turbinaria ornate*<br>Hot water extracts	Antioxidant<br>Free-radical scavenging	[129]
Fucoidans	Brown macro algae<br>*Undaria pinnatifida,*<br>*Fucus vesiculosus*<br>Extracts	Topical benefits<br>(comparative *in vitro*<br>and double-blind,<br>placebo-controlled clinical studies)	[130]
Four fucoidan fractions<br>(fucose, rhamnose, xylose, mannose, glucose, and galactose<br>in different mole ratios)	Brown seaweed<br>*Sargassum pallidum*<br>(Yelow sea)<br>Water extracts	Pharmaceutical resource<br>Functional food	[131]
Fucoidans	Brown seaweeds<br>*Sargassum ilicifolium,*<br>*Sargassum angustifolium*<br>(Qeshm Island, Iran)<br>Extracts	Antioxidant<br>Antimicrobial<br>Nutraceutical industry	[40]
Sulphated polysaccharides	brown algae<br>*Padina boryana*<br>(Maldives)<br>Extracts<br>(celluclast enzyme assisted extraction;<br>ethanol precipitation)	Antioxidant<br>(*in vitro* and in<br>Zebrafish model *in vivo*)	[132]

Substances with antioxidant activity	Origin	Biological activity<br>Potential applications	Ref.
Fucoidan<br>(sulfated polysaccharides<br>and small amounts<br>galactose, xylose, glucose, and mannose)	Brown seaweed<br>*Sargassum filipendula*<br>Extracts	Free radical scavenging<br>(dependent on the extraction<br>method, temperature, time<br>and solvent concentration)	[133]
Α-Carrageenan	Red algae<br>*Saccharina japonica*<br>Extracts	Anti-coronaviruses	[138]<br>[139]<br>[140]
New sulphated polysaccharides	Red algae<br>*Gracilaria caudate (SP-Gc)*<br>Enzymatic extraction	Antioxidant<br>Hydrocolloids	[141]
Sulphated polysaccharides,<br>(molar ratio galactose:3,6-anhydro-galac-<br>tose = 1.2:1.0; sulphate content about 3.8%)	Alga<br>*Porphyra haitanensis*	Radical scavenging	[142]
**Therpens**			
New scalarane-type<br>sester-terpenes:<br>hyrtioscalarane A<br>hyrtioscalaren B	Demosponge<br>*Hyrtios erectus*<br>Organic extracts	Antioxidant<br>Anti-inflammatory<br>(selectivity index<br>higher than that<br>of Ibuprofen)	[143]
Monoterpenoid (−)-Loliolide	seaweed<br>*Sargassum horneri*	Antioxidant<br>(at Vero cells and<br>in Zebrafish models)	[144]
Thyrsiferol	red algae<br>genus *Laurencia*	Anti-viral<br>Anti-tumour	[49]
Carotenoid<br>Astaxanthin (red pigment)	-	Antioxidant<br>(10-fold greater than<br>other carotenoids)	[146]
Saproxanthin<br>Myxol<br>(rare marine monocyclic carotenoids)	Bacteria<br>family *Flavobacteriaceae*	Antioxidant<br>(stronger than of<br>Zeaxanthin and β-carotene)	[147]
**Polyphenols**			
Bromophenols<br>Phenolic acids<br>Flavonoids<br>Florotannins	-	Antioxidant<br>(on different mechanisms)<br>Singlet oxygen and<br>free radicals scavenging<br>Chelating	[151]
Ovothiols<br>(thiol histidine derivatives)	Synthesized<br>by two enzymes:<br>sulfoxide synthase OvoA,<br>sulfoxide lyase Ovo B.	Unusual antioxidant<br>Anti-proliferative<br>Anti-fibrotic	[153]
**Enzymatic antioxidants**			
Recombinant superoxide dismutase<br>metallo-enzyme	Pacific abalone<br>*Haliotis discus hannai Ino*<br>*(P. pastoris)*	Antioxidant<br>Endogenous,<br>Exogenous<br>free radicals scavenging	[155]
New digestive α-amylase	Blue Crab<br>*Portunus segnis*<br>Viscera	New features<br>(compared to other<br>marine-derived enzymes)	[157]

Substances with antioxidant activity	Origin	Biological activity<br>Potential applications	Ref.
		Oat flour antioxidant potential	
